# Improvement of Eye Alignment in Adult Strabismic Monkeys by Sustained IGF-1 Treatment

**DOI:** 10.1167/iovs.16-19739

**Published:** 2016-11

**Authors:** Linda K. McLoon, Stephen P. Christiansen, Geoffrey M. Ghose, Vallabh E. Das, Michael J. Mustari

**Affiliations:** 1 Department of Ophthalmology and Visual Neurosciences, University of Minnesota, Minneapolis, Minnesota, United States; 2 Department of Neuroscience, University of Minnesota, Minneapolis, Minnesota, United States; 3 Departments of Ophthalmology and Pediatrics, Boston University School of Medicine, Boston, Massachusetts, United States; 4 College of Optometry, University of Houston, Houston, Texas, United States; 5 Washington National Primate Center and Department of Ophthalmology, University of Washington, Seattle, Washington, United States

**Keywords:** insulin growth factor-1, myogenic growth factors, eye movements, extraocular muscles, strabismus, innervation, smooth pursuit, neuromuscular junctions

## Abstract

**Purpose:**

The goal of this study was to determine if continuous application of insulin-like growth factor-1 (IGF-1) could improve eye alignment of adult strabismic nonhuman primates and to assess possible mechanisms of effect.

**Methods:**

A continuous release pellet of IGF-1 was placed on one medial rectus muscle in two adult nonhuman primates (M1, M2) rendered exotropic by the alternating monocular occlusion method during the first months of life. Eye alignment and eye movements were recorded for 3 months, after which M1 was euthanized, and the lateral and medial rectus muscles were removed for morphometric analysis of fiber size, nerve, and neuromuscular density.

**Results:**

Monkey 1 showed a 40% reduction in strabismus angle, a reduction of exotropia of approximately 11° to 14° after 3 months. Monkey 2 showed a 15% improvement, with a reduction of its exotropia by approximately 3°. The treated medial rectus muscle of M1 showed increased mean myofiber cross-sectional areas. Increases in myofiber size also were seen in the contralateral medial rectus and lateral rectus muscles. Similarly, nerve density increased in the contralateral medial rectus and yoked lateral rectus.

**Conclusions:**

This study demonstrates that in adult nonhuman primates with a sensory-induced exotropia in infancy, continuous IGF-1 treatment improves eye alignment, resulting in muscle fiber enlargement and altered innervational density that includes the untreated muscles. This supports the view that there is sufficient plasticity in the adult ocular motor system to allow continuous IGF-1 treatment over months to produce improvement in eye alignment in early-onset strabismus.

Strabismus has been treated in much the same way for more than 100 years.^1^ If patching and/or spectacle wear fails to restore normal eye alignment, strabismus surgery is the next treatment approach. This involves surgical manipulation of the extraocular muscle (EOM) insertions on the sclera. However, 20% to 50% of such surgeries fail to produce good alignment (defined as residual misalignment of <10 prism diopters).^2–6^ Long-term failure rates for strabismus surgery are even higher; in one study, the 10- and 20-year failure rate was 51% and 66%, respectively, defined as those subjects requiring a second surgery.^7^ In a second study, failure rates were 86% by 15 years.^8^ These longitudinal studies suggest that surgical correction of eye position does not adequately address the underlying cause of the misalignment, which, in turn, drives return of the strabismus.

One possible explanation is that the visual system may be unable to adapt to abrupt changes in alignment produced by surgery. As a consequence, other visual or oculomotor adaptive processes act to gradually return the eyes to the presurgical state of misalignment. If this is the case, then more gradual and sustained treatment that potentiates muscle and nervous system plasticity may offer a solution. This hypothesis forms the basis of the present experiment.

Insulin-like growth factor (IGF-1) is a neurotrophic factor known to have a role in muscle growth during development and regeneration.^9,10^ It is able to increase muscle size and improve function when upregulated in the muscles of mouse models of muscular dystrophy and in sarcopenia associated with aging.^11,12^ In a series of studies in adult rabbit EOMs, we demonstrated that single injections and sustained delivery of IGF-1 resulted in significant increases in the size and force generation in the treated rectus muscles.^13,14^ After single injections in chicks^15^ and sustained delivery in infant monkeys,^16,17^ IGF-1 treatment also resulted in larger myofibers.

These results suggested that sustained delivery of IGF-1 has potential as an effective long-term treatment for strabismus. Sustained delivery of IGF-1, and the potential slow and gradual changes associated with such delivery, would avoid the large single change in eye alignment often associated with surgical intervention and, therefore, allow plasticity mechanisms in the visual system and/or ocular motor system to adapt to the change in eye alignment.^18–21^ This conceptually has interesting implications for strabismus treatment, and experimental animal models allow this to be studied in more detail.

As a proof of concept, two adult macaque monkeys at age 6 years, made exotropic using the alternating monocular occlusion method in infancy,^22,23^ were implanted with sustained release IGF-1 pellets unilaterally. Fixation and smooth pursuit movements were assessed periodically for 3 months. At the end of the 3-month period, which is the duration of IGF-1 release from the implanted pellets, the horizontal extraocular muscles of one monkey (M1) were examined morphometrically for changes in myofiber size and density of innervation. Despite their long-term strabismus, both monkeys showed improvement in eye alignment. While the improvement of M1 was superior to the result in the second monkey, this study demonstrated that sustained release of neurotrophic factors can alter eye alignment bilaterally in adult strabismus after a unilateral treatment, even when the strabismus was induced by sensory deprivation in infancy.

## Methods

### Animals and Surgery for Sustained IGF-1 Treatment

All studies were approved by the animal care and use committees at Emory University and the University of Minnesota, and all experiments adhered to the National Institutes of Health (NIH; Bethesda, MD, USA) Animal Care and Use Guidelines and ARVO Guidelines for Use of Animals in Ophthalmic and Vision Research. Adult monkeys were used as the source of experimental data and for all the tissues analyzed in this study.

Within the first 24 hours after birth, two infant monkeys (*Macaca mulatta*; M1, M2) were treated daily with an opaque contact lens placed on one eye for 24 hours, switched to the fellow eye for the next 24 hours, and this patching was alternated on a daily basis for a period of 4 months. The patched infant monkeys were monitored 8 to 12 times per day every day to ensure that the contact lens remained in place. The lack of binocular input produced exotropia in both monkeys, and both were alternating monocular fixators. At approximately 3 years of age, the monkeys were implanted with a head stabilization post and scleral search coils in each eye for precise eye movement measurement. All surgeries were performed using sterile technique under isoflurane anesthesia (1.25%–2.5%).

The animals were trained to perform standard eye movement tasks, including fixating stationary visual targets at known eccentricities, visually guided saccades, and smooth pursuit of moving targets (±10°–20°; 0.25–0.5 Hz) projected on a tangent screen. At the end of these experiments, approximately, 6 to 8 years, the monkeys were selected for IGF-1 treatment. Under general anesthesia using standard sterile technique, an incision was made in the medial conjunctiva, and after visualization of the medial rectus muscles, a 3 × 1.5-mm pellet prepared to release 1 μg/d of IGF-1 for a total of 90 days (Innovative Research of America, Sarasota, FL, USA) was implanted on the orbital side of a single medial rectus muscle in each monkey. This dose was selected based upon previous studies.^13^ After pellet implantation, the conjunctiva was closed with an 8-0 polyglactin suture. At 2 months after pellet implantation, verification of pellet position in the first monkey was performed by magnetic resonance imaging (MRI; axial sequence; 3T Siemens Trio 3-Tesla magnetic resonance imager, *T*_1_-weighted fast spin-echo; Siemens, Munich, Germany).

To examine the effect of IGF-1 on alignment and eye movements, binocular eye and target data were collected as the animals performed a fixation task – fixating a series of targets along the horizontal or vertical meridian, and a smooth-pursuit task – tracking of sinusoidal motion of target (0.3 Hz ± 10°–15°) along the horizontal plane under monocular, right eye, or left eye viewing conditions. Binocular eye position was measured using the magnetic search coil technique (Angle-Meter; Primelec Industries, Regensdorf, Switzerland) as described previously.^23^ This system provides precise and reliable measurement of eye position to a resolution better than 0.5°. Eye coil signals were calibrated under monocular viewing conditions by rewarding the monkey for looking within a 2° to 3° window surrounding a 1° target that was rear projected on a tangent screen 60 cm away from the animal. Visual stimuli were generated under computer control using a VSG2/5 visual stimulus generator installed in a Windows PC (Cambridge Research Systems Ltd, Rochester, England). Binocular eye and target position feedback signals were processed with anti-aliasing filters at 400 Hz before digitization at 1 KHz with 16-bit precision (Data Acquisition boards and Labview software; National Instruments, Austin, TX, USA). Data analysis was performed with custom software routines written in Matlab (Mathworks, Natick, MA, USA) and involved examining alignment of the eyes during fixation and smooth pursuit eye movements.

At the end of 3 months, M1 was deeply anesthetized, followed by euthanasia performed by a licensed veterinarian. All medial and lateral rectus muscles were dissected in their entirety from origin to insertion, embedded in tragacanth gum and rapidly frozen in 2-methylbutane chilled to a slurry on liquid nitrogen. The muscles were stored at −80°C. In addition, three sets of naïve control EOM were processed similarly. These muscles were collected after euthanasia from 3 adult monkeys (*M. mulatta*) that had been used for nonrelated studies at the University of Minnesota. None of these animals had strabismus nor were subjected to EOM manipulations or treatments. Previous studies showed that there were no measurable changes in the vertical muscles, so they were not analyzed for the present study.^16,17^

Muscles were sectioned at 12 μm using a Leica cryostat (Leica; Wetzlar, Germany), and the slides were air-dried and then stored at −80°C until processed. Every 20th slide was stained with hematoxylin and eosin. A second set was immunostained for the fast myosin heavy chain isoform (1:20; Vector Laboratories, Burlingame, CA, USA), neurofilament protein (smi-31, 1:1000; Covance, Princeton, NJ, USA), or for the neuromuscular junction using α-bungarotoxin labeled with Alexa Fluor 488 (1:3000; Molecular Probes, Eugene, OR, USA). For mean cross-sectional area, a minimum of 3 slides from the mid belly region and 3 slides toward the tendon end were analyzed, with a minimum of 200 myofibers measured per slide from the orbital and global layers. For nerve density, again 3 sections from the tendon and mid belly regions were analyzed for nerve density as a percentage of overall muscle area using the Bioquant Morphometry System (Bioquant, Nashville, TN, USA) as described previously.^17^ Briefly, nerves and total area were quantified from a minimum of 3 fields in orbital and global layers in the mid belly and tendon regions to yield density of nerves per cross-sectional area. Neuromuscular junction density was determined similarly, and reported as number per cross-sectional area. Sizes of neuromuscular junctions also were determined in control and IGF-1–treated medial rectus muscles, either as area or length, and then analyzed relative to myofiber area or perimeter, respectively. All neuromuscular junctions on 3 cross-sections were photographed, and measured using the Bioquant system. The large number of myofibers and neuromuscular junctions examined allowed the use of a 1-way ANOVA followed by a Tukey's multiple comparison post hoc test to determine significance, where *P* < 0.05. Data are presented at mean ± SEM.

## Results

**Figure 1:**
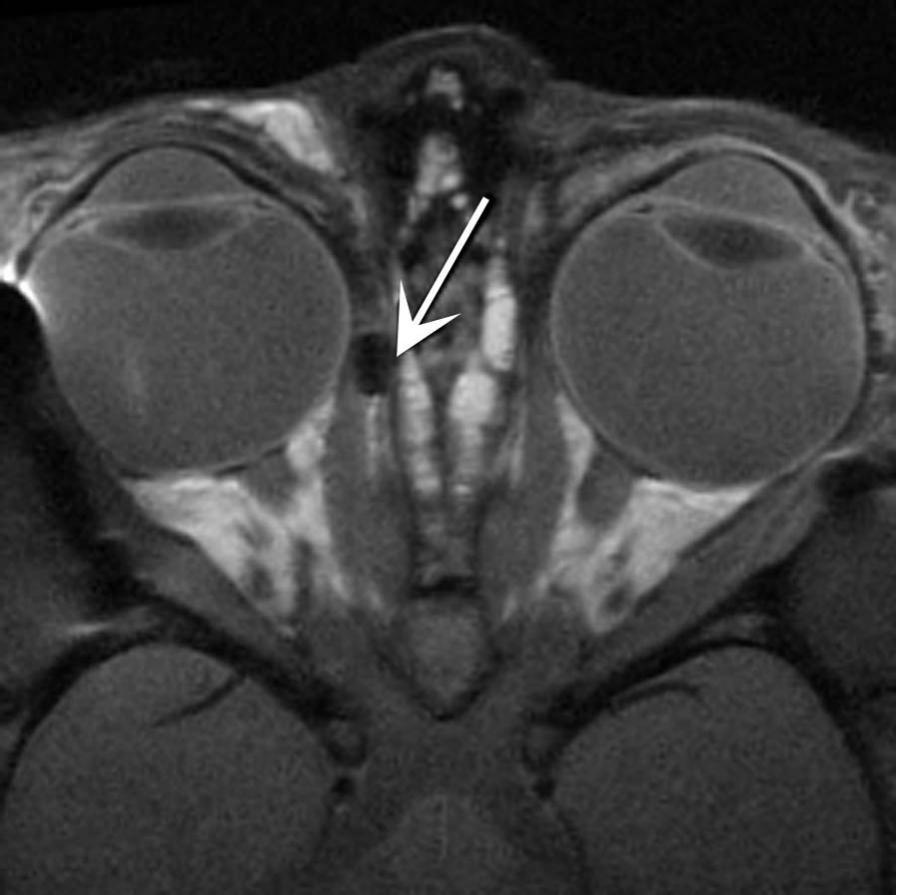
Magnetic resonance imaging (MRI) of M1 at 2 months showing the pellet in place on the medial rectus muscle (*arrow*) performed using a Siemens 3-T imager, *T*_1_-weighted fast spin-echo.

**Figure 2:**
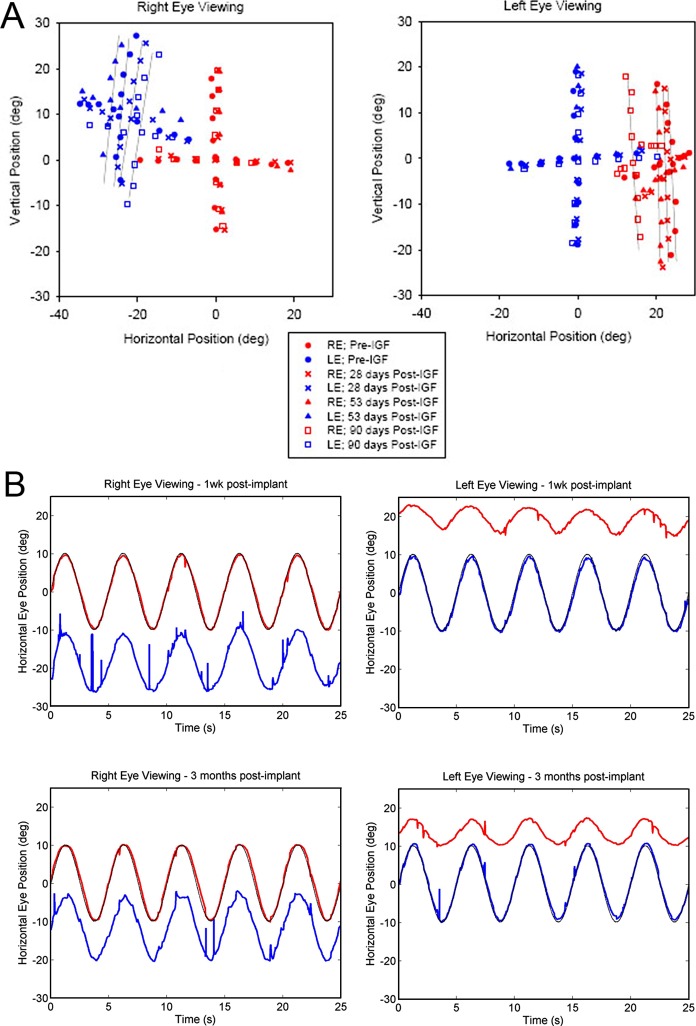
(**A**) Eye alignment of M1 showing the slow improvement in eye alignment over the first 2 months, and a large change in strabismus angle during month 3 of continuous IGF-1 treatment. (**B**) Smooth pursuit movements in M1 showing the improvement in eye alignment between the initial time point and after 3 months of IGF-1 treatment. *Red* represents the position of the right eye, *blue* the position of the left eye, and *black* represents the target position; +ve values indicate rightward eye positions and –ve values are leftward eye positions.

At the time of pellet implantation, both monkeys were exotropic. Pellet location was confirmed with MRI in M1 (Fig. 1). The pellet in M2 could not be definitely identified on the muscle with MRI at 2 months. The initial angle of misalignment of M1 was approximately 28° with the right eye viewing and approximately 25° with the left eye viewing (Fig. 2A). During the first 2 months of pellet treatment, the angle of misalignment changed very slightly, from 28° to 24° in M1 (Fig. 2A). At the end of the third month, M1 showed a large improvement in eye alignment; the strabismic angle changed to 18° in the right eye, an improvement of 10°, and to 14° in the left eye, an improvement of 11° (Fig. 2). This represents an improvement in strabismic angle of 35.7% and 44%, respectively. This also can be seen on the smooth pursuit data (Fig. 2B). It should be noted that these changes occurred bilaterally, despite only one medial rectus muscle being treated.

**Figure 3:**
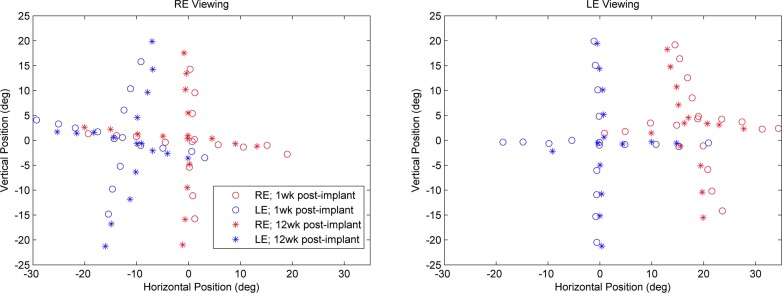
Eye alignment of M2 showing change in eye alignment from 1 week after implant and 3 months after the start of the IGF-1 treatment period. *Red* represents the position of the right eye and *blue* the position of the left eye.

For M2 the initial angle of misalignment was approximately 12.50° with the right eye viewing and approximately 19° with the left eye viewing (Fig. 3). Monkey 2 also showed an improvement in eye alignment, 2.5° in the right eye and 3° in the left eye (Fig. 3). This represents an improvement in strabismic angle of 20% and 15.8%, respectively. The MRI at 2 months could not locate the pellet placement definitively, and this may explain the differential in effectiveness seen between the two treated monkeys.

**Figure 4:**
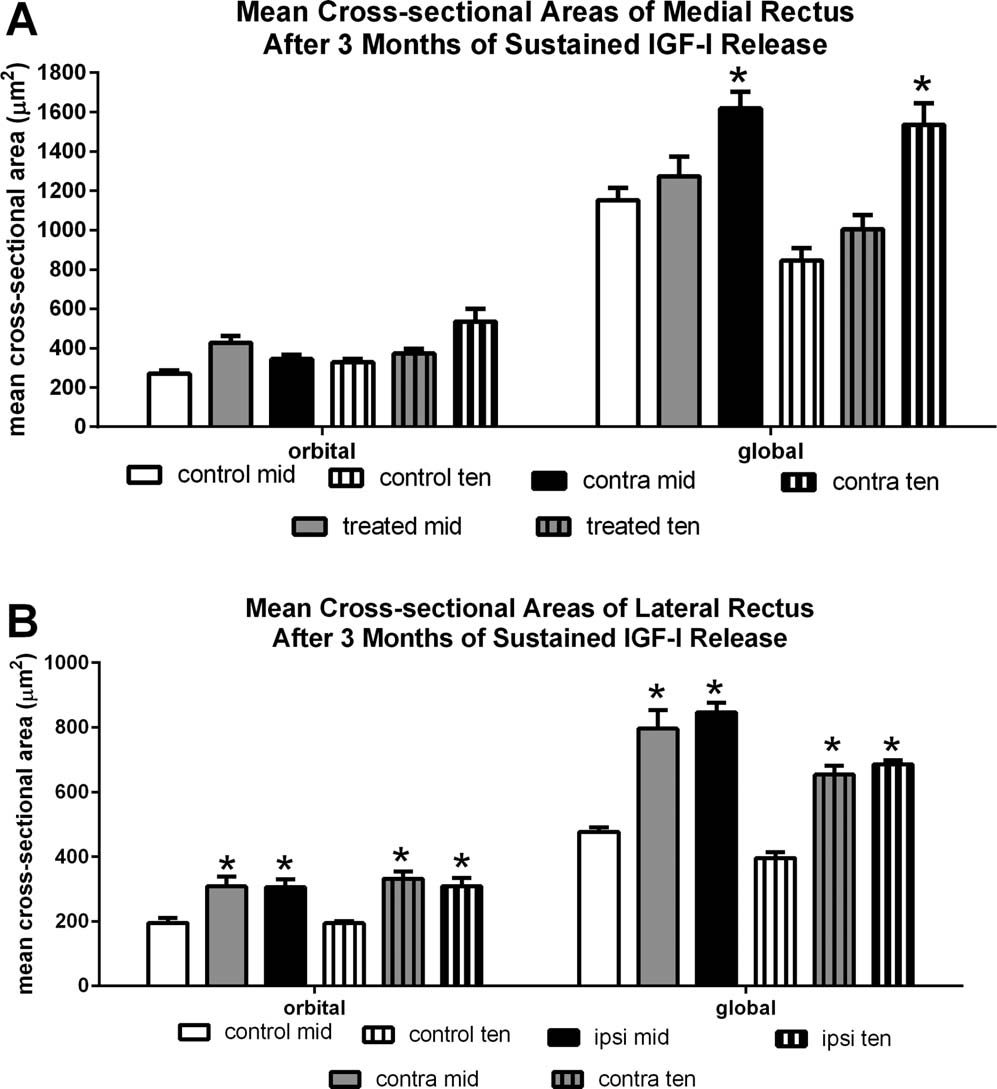
(**A**) Mean cross-sectional areas of the medial rectus muscles from controls and M1 after the 3-month IGF-1 treatment period. (**B**) Mean cross-sectional areas of the lateral rectus muscles from controls and M1 after the 3-month IGF-1 treatment period. *Significant difference from naïve control myofiber population in the same layer and muscle region.

To better understand the structural changes that caused this change in eye alignment, mean cross-sectional areas of the EOM were determined for M1. The mean myofiber cross-sectional area of the treated muscle was consistently larger than the naïve control muscles (Fig. 4). For the midbelly region, after the 3 months of sustained IGF-1 treatment the orbital layer fibers were 57.8% larger than control, at 427.61 ± 35.4 μm^2^ compared to 270.99 ± 15.25 μm^2^, and the global layer fibers were 11% larger than control myofibers, 1272.96 ± 101.95 μm^2^ compared to 1151.32 ± 64.48 μm^2^ (Fig. 4A). Differences were similar in the tendon region, which was closer to the pellet. The mean cross-sectional areas for the treated orbital fibers in the tendon region were 13.4% larger, 272.84 ± 24.74 μm^2^ compared to the treated muscle at 328.88 ± 17.09 μm^2^, and the treated global fibers were 19% larger, 1003.45 ± 74.88 μm^2^ compared to 844.46 ± 64.64 μm^2^ (Fig. 4A). The contralateral untreated medial rectus also showed an increase in mean myofiber cross-sectional areas, particularly in the tendon region. The mean cross-sectional areas of the untreated medial rectus muscle on the contralateral side were 27% and 41% larger in the orbital and global layers in the mid belly region than control areas, and 60% and 82% larger in the orbital and global layers in the tendon region compared to control values. Additionally there were changes in the yoked lateral rectus muscle of the contralateral orbit, with increases in the middle region of 58% and 44%, and increases in the tendon region of 71% and 18% for the orbital and global layers respectively. Similarly, changes were seen in the lateral rectus muscle ipsilateral to the treated medial rectus muscle. These types of increases were seen in the untreated muscles of infant monkeys made strabismic after 3 months of unilateral IGF-1^17^ or GDNF treatment (McLoon L, et al. *IOVS* 2016;57:ARVO E-Abstract 1395). Note that the lateral rectus myofibers were significantly smaller than the medial rectus myofibers in the control monkeys.

**Figure 5:**
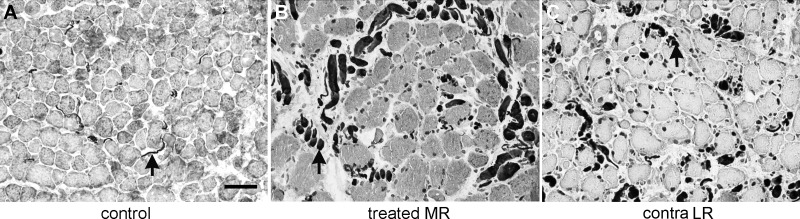
Immunostaining for neurofilament protein (smi-31) in controls and M1 in (**A**) an aged-matched control monkey medial rectus muscle, (**B**) the treated medial rectus muscle, and (**C**) the yoked lateral rectus muscle in the contralateral orbit. *Arrows* indicate nerves within the muscles. *Scale bar*: 50 μm.

**Figure 6:**
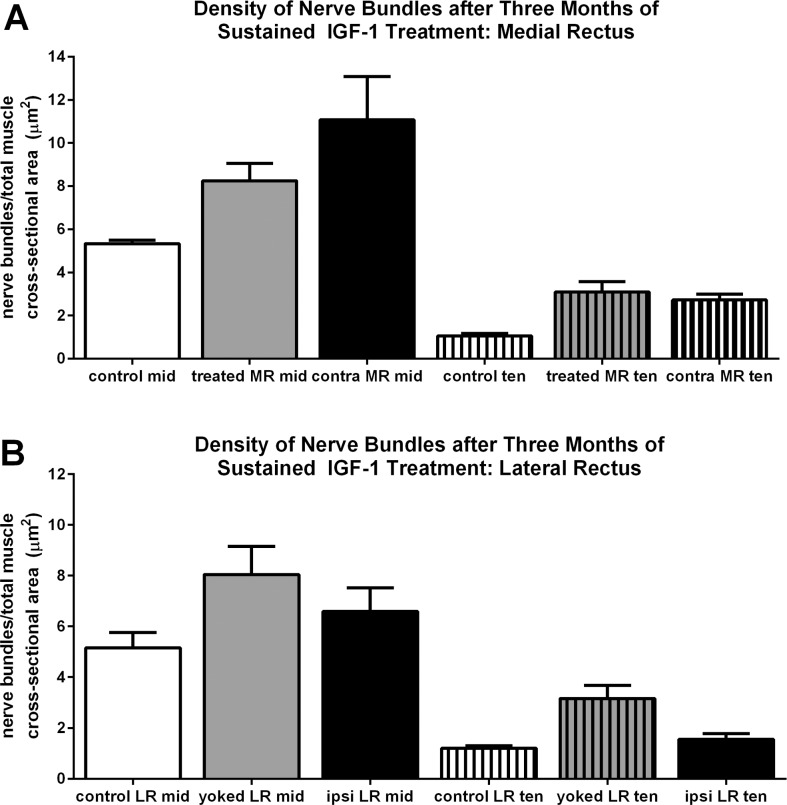
Quantification of nerve density in controls and M1 in the middle of the muscle and in the tendon region (**A**) in the medial rectus muscles and in the (**B**) lateral rectus muscles. Ten, tendon; mid, mid region; contra, contralateral; ipsi, ipsilateral. *Significant difference from naïve control muscles.

Insulin-like growth factor-1 has multiple actions in skeletal muscle. In addition to causing myofiber hypertrophy, it also can increase nerve outgrowth.^24,25^ Using an antibody to neurofilament protein, the overall density of nerve fibers was assessed based on total cross-sectional area (Figs. 5, 6). There was a pronounced increase in nerve fiber density in the treated medial rectus muscle compared to control specimens (Figs. 5, 6A). In the midregion of the muscles, the treated muscle nerve density was 55% greater, 8.25 ± 0.81 compared to 5.33 ± 0.16 μm^2^. In the tendon region, there was a 192% increase in nerve density, 3.1 ± 0.48 μm^2^ compared to the control at 1.06 ± 0.12 μm^2^. In the untreated, contralateral medial rectus, there was a large increase in nerve density compared to the control muscles, with increases of 108% in the midregion and 158% in the region near the tendon. This represents, respectively, an increase of 34% and a decrease of 11% in the middle and tendon regions compared to the treated medial rectus muscle. Increases in the innervation of the yoked lateral rectus muscle also were seen.

**Figure 7:**
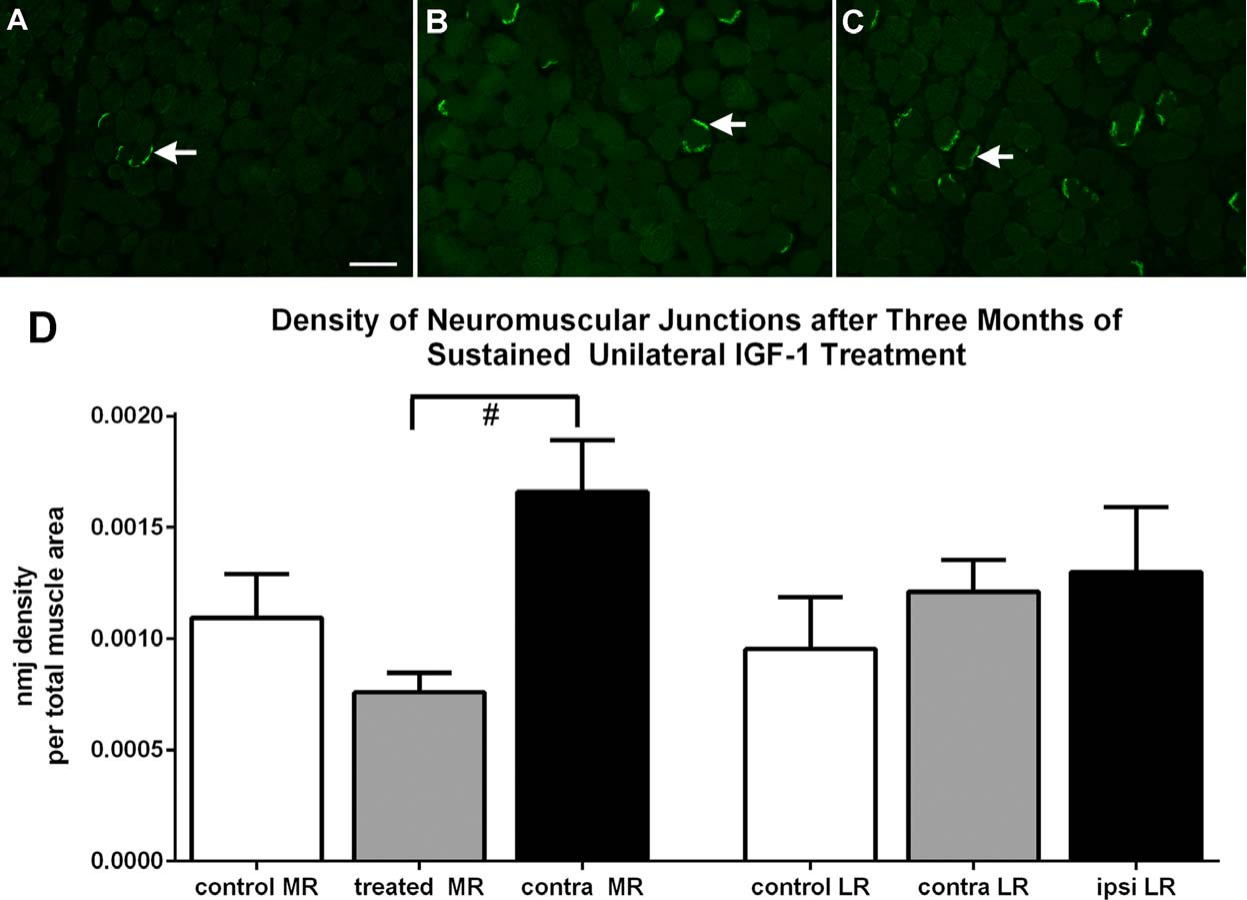
Visualization of the neuromuscular junctions with fluorescently labeled α-bungarotoxin in (**A**) the control monkey, and in M1 (**B**) the treated medial rectus, and (**C**) the contralateral medial rectus muscle. *Arrows* indicate neuromuscular junctions. *Scale bar*: 75 μm. (**D**) Quantification of neuromuscular junction density in all medial and lateral rectus muscles in controls and M1. #Significant difference compared to the treated medial rectus muscle.

An increase in nerve density should translate into changes in neuromuscular junction density or size. Interestingly, analysis showed that there was a 31% lower density of neuromuscular junctions in the treated compared to the control medial rectus muscles, 0.00007586 ± 0.00005/μm^2^ compared to control at 0.001094 ± 0.0002/μm^2^, but a very large 52% increase in the density of the neuromuscular junctions in the contralateral medial rectus muscle at a density of 0.00166 ± 0.0002/μm^2^ (Fig. 7). In addition, compared to normal control lateral rectus muscles, there was approximately a 25% to 57% increase in neuromuscular junction density in the yoked and ipsilateral lateral rectus muscles.

**Figure 8:**
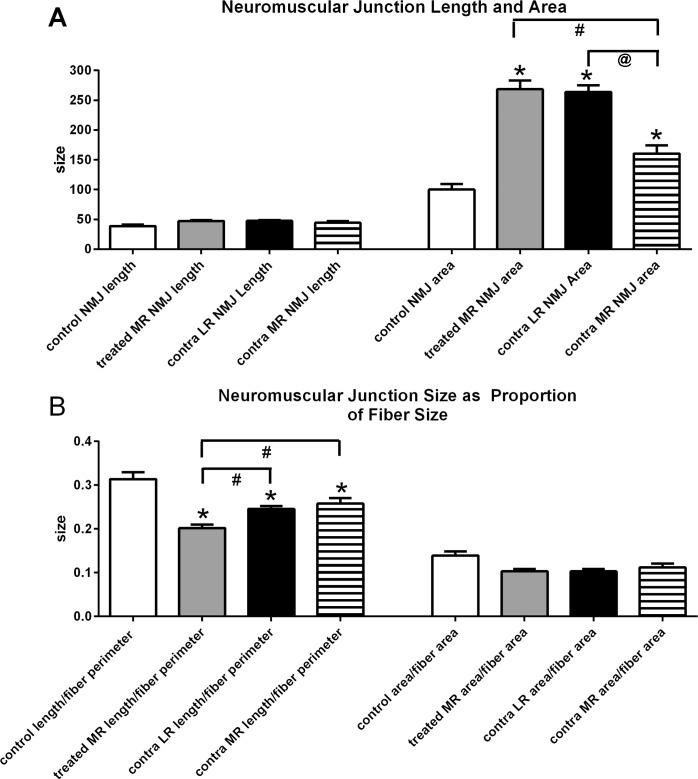
(**A**) Average neuromuscular junction (NMJ) length and area. (**B**) Average size of NMJs calculated as length/fiber perimeter and area/fiber area in control medial rectus muscles (MR), in the IGF-1–treated MR, contralateral MR, and yoked lateral rectus muscle (LR) in the contralateral orbit. *Significant difference from naïve control muscles. #Significant difference compared to the treated medial rectus muscle. @Significant difference between contra LR and contra MR.

Neuromuscular junction length (μm) and area (μm^2^) were assessed in control medial and lateral rectus muscles, and in all four of the horizontal rectus muscles in M1 (Fig. 8). There were no alterations in the mean neuromuscular junction length in any of the muscles examined. However, there was a large 168% increase in the mean neuromuscular junction area in the treated medial rectus muscle compared to controls, an increase of 60% in the contralateral medial rectus muscle, and an increase of 163% in the yoked lateral rectus muscle (Fig. 8A). As mean fiber size was altered by the IGF-1 treatment, neuromuscular junction size was reanalyzed either as length as a proportion of myofiber perimeter or area as a proportion of myofiber area (Fig. 8B). In all the muscles analyzed from M1, the ratio of neuromuscular junction length to myofiber perimeter was decreased compared to control muscles, from 37% for the treated medial rectus muscle to 18% to 22% for the medial and lateral rectus muscles in the contralateral orbit (Fig. 8B). This analysis demonstrated that relative to fiber perimeter, the neuromuscular junctions in horizontal muscles of the IGF-1 treated monkey were 20% to 26% smaller than controls. In summary, IGF-1 treatment in the adult strabismic monkey resulted in an increased density of neuromuscular junctions, but they were proportionally smaller than those in age-matched control medial rectus muscles.

## Discussion

Three months of sustained release treatment with IGF-1 adjacent to a single medial rectus muscle improved the eye alignment of adult nonhuman primates with a sensory-induced exotropia created in infancy. Myofiber hypertrophy is a known effect of IGF-1 treatment, particularly when it is maintained over time as was done in the present study.^10,12,26^ In M1, which had the most significant improvement in eye alignment, the mean cross-sectional areas of the treated myofibers were enlarged by 11% to 58% in the midbelly region and 13% to 19% in the tendon region compared to age-matched control medial rectus muscles. Despite only treating one medial rectus muscle, increased mean cross-sectional areas also were evident in the medial rectus muscle in the contralateral orbit, with increases of 27% and 41% in the mid belly region and 60% to 82% in the tendon region. Similarly there were increases in mean myofibers areas in the functionally yoked lateral rectus muscle. It is interesting that the largest increases in myofiber area were in the untreated medial rectus on the contralateral side. This suggested that part of the bilateral improvement in eye alignment after unilateral IGF-1 treatment is related to the ability of the oculomotor system to estimate muscle size and force production, and drive compensation of the muscles bilaterally. Our previous work demonstrated that unilateral, but not bilateral, medial rectus muscle treatment with IGF-1 in infant primates was required to produce a strabismus also suggesting central nervous system control of these changes.^17^

There was increased nerve density as a result of the IGF-1 treatment, another well described function of muscle-derived IGF-1.^24,25^ Insulin-like growth factor-1 has been shown to cause increased axon number, size, and density when locally delivered.^27^ Thus, the increased nerve density in the treated medial rectus muscle was expected. It is noteworthy that there was significant nerve growth into the untreated medial rectus muscle in the contralateral orbit; this type of change in untreated muscles is a common feature of unilateral treatments of the EOM.^28,29^ There also was increased nerve density in the contralateral lateral rectus muscle. Such coordinated adaptations of untreated muscles that are contralateral to a given treatment have been described in the exercise physiology literature. Resistance exercise has been shown to result in increased expression of IGF-1, demonstrating the effects of elevated IGF-1 when levels were increased unilaterally.^29^ For example, in one study, 4 weeks of unilateral resistance training resulted in significant increases in muscle strength on the untrained side.^30^ In a study in stroke patients, high intensity unilateral resistance training of one limb resulted in significant gains in force in the contralateral but nonexercised limb.^31^ Using a transcranial magnetic stimulation method, unilateral training protocols were shown to reduce intracortical inhibition and silent period duration as well as increase corticospinal excitability.^32,33^ The physiologic bases for these changes are not understood, but our data support the view that there is bidirectional communication between the central brain areas and yoked and agonist-antagonist muscle pairs. As indicated, it is important to remember that resistance exercise resulted in increased muscle expression of IGF-1.^34,35^ Thus, the unilateral resistance training was able to induce significant changes of innervation in the untreated muscles of the contralateral side, similar to what we observed in the present experiment, at least in part by an increase in IGF-1.

Nerve density does not necessarily equate to changes in neuromuscular junction density. However, it is known that IGF-1 also can cause increased postsynaptic density size and complexity^36^ as well as synaptic potentiation.^37^ This is interesting in light of the apparent decrease in neuromuscular junction density in the treated medial rectus muscle, although it was not significantly different from naïve control neuromuscular junction density. There was, however, a very large increase in neuromuscular junction density in the untreated medial rectus muscle in the contralateral orbit, which correlated very well with the significant increase in nerve density. Neuromuscular junction density was unchanged in the lateral rectus muscles. In addition to number of neuromuscular junctions per muscle area, the complexity of neuromuscular junctions can be affected by IGF-1.^36^ We calculated the average size and area of neuromuscular junctions and in addition determined their size relative to myofiber perimeter and area, respectively. The average neuromuscular junction area significantly increased in the treated medial rectus and the contralateral yoked lateral rectus muscle. However, when examined relative to myofiber size, the story is more complicated. There were small decreases in the length of the neuromuscular junctions in the treated medical rectus muscle and contralateral lateral rectus muscle in proportion to fiber perimeter, while the neuromuscular junctions were significantly larger in the contralateral medial rectus muscle than in the treated muscle. This suggested that after 3 months of adaptation to exogenously applied IGF-1 to a single medial rectus muscle, there was an increased drive from the innervating motor neurons of the untreated medial rectus. The method for communication of single muscle changes within the brain is not known. At the end of the 3 months of exogenous IGF-1 treatment, there also appeared to be a reduced drive from the motor neurons innervating the hypertrophied treated medial rectus muscle to reduce its “force” by decreasing innervation to this muscle. This hypothesis correlated with neuronal recordings after resection/recession surgery, which demonstrated significant changes in neuronal drive 1 to 6 months after surgery correlating with worsening strabismic angle (Pullela M, et al. *IOVS* 2015;56:ARVO E-Abstract 5221; Agaoglu MN, et al. *IOVS* 2015;56:ARVO E-Abstract 5222). In other words, in addition to moment-by-moment changes in innervation to the untreated eye that is a normal response to changes in yoked muscle contractility of the treated eye, there also are longer term adaptive processes that actually change muscle structural properties and neuromuscular junction structural properties of the untreated eye.^28,38,39^ Both of these factors influenced misalignment when the subjects viewed with their treated eye. With respect to synaptic efficacy, it remains to be determined whether these synaptic changes are accompanied by fine-scale structural changes within the synapse, such as a change in the size or efficacy of active zones in the presynaptic elements.^40–42^

Insulin-like growth factor-1 has been implicated in restoring synaptic plasticity in the mature brain, including sensorimotor^43^ and visual cortex.^44^ Our observations suggested that sustained release IGF-1 unilaterally also has a similar potential to modulate neuromuscular changes at the muscle level, presumably driven by the oculomotor system at the brainstem level. It has been demonstrated clearly that brain activity, as opposed to muscle changes alone, contribute to the control of strabismus angle.^45–47^ In cat abducens motor neurons, it also has been shown that various neurotrophic factors can regulate the firing patterns and synaptic composition of the motor neurons that control eye movements.^48,49^ These studies support the hypothesis that neurotrophic factors are modulating influences on motor neuron function, and by altering the balance of these factors one has the potential to alter the balance of eye alignment/misalignment through communication between oculomotor and abducens motor neurons within the brainstem. The bilateral nature of the changes in alignment and EOM structure in the present study, after unilateral IGF-1 treatment, provides further support for this view. Previous studies in rabbit showed that IGF-1 localization was dramatically altered and moved from the inside of the fiber to its periphery after experimental strabismus surgery in rabbits.^28^ This strongly implicates the potential extracellular release of IGF-1 in myofiber remodeling and regenerative processes. While our studies of continuous glial cell line-derived neurotropic factor (GDNF) treatment of one medial rectus muscle in infants resulted in both induction of strabismus as well as maintenance of strabismus after treatment was completed (McLoon L, et al. *IOVS* 2016;57:ARVO E-Abstract 1395), it remains to be determined whether these improvements to eye alignment after sustained IGF-1 treatment in adult strabismic monkeys would be maintained. Our study suggests that targeted and sustained neurotrophic factor therapies, which leverage inherent oculomotor plasticity mechanisms, have the potential to augment current surgical approaches to provide lasting solutions to cure strabismus.
